# Vitamin C Supplementation for the Treatment of COVID-19: A Systematic Review and Meta-Analysis

**DOI:** 10.3390/nu14194217

**Published:** 2022-10-10

**Authors:** Monika Olczak-Pruc, Damian Swieczkowski, Jerzy R. Ladny, Michal Pruc, Raul Juarez-Vela, Zubaid Rafique, Frank W. Peacock, Lukasz Szarpak

**Affiliations:** 1 ViaMed Polyclinic, 02-495 Warsaw, Poland; 2 Department of Toxicology, Faculty of Pharmacy, Medical University of Gdansk, 80-210 Gdańsk, Poland; 3 Department of Emergency Medicine, Medical University of Bialystok, 15-089 Bialystok, Poland; 4 Research Unit, Polish Society of Disaster Medicine, 05-806 Warsaw, Poland; 5 Faculty of Health Science, University of La Rioja, Logroño, 26006 La Rioja, Spain; 6 Henry JN Taub Department of Emergency Medicine, Baylor College of Medicine, Houston, TX 77030, USA; 7 Institute of Outcomes Research, Maria Sklodowska-Curie Medical Academy, 00-136 Warsaw, Poland

**Keywords:** vitamin C, Ascorbic Acid, SARS-CoV-2, COVID-19, Corona Virus Disease 2019, meta-analysis, hospital mortality, length of stay

## Abstract

Since the outbreak of the coronavirus disease 2019 (COVID-19) pandemic, caused by the severe respiratory syndrome coronavirus 2 (SARS-CoV-2), millions of people have died, and the medical system has faced significant difficulties. Our purpose was to perform a meta-analysis to estimate the effect of vitamin C on in-hospital mortality and the ICU or hospital length of stay for patients diagnosed with COVID-19. We conducted a systematic review with meta-analysis in the following databases: PubMed, Web of Science, Scopus and Cochrane Central Register of Controlled Trials. We included studies that evaluated the effect of vitamin C supplementation, compared with standard treatment in COVID-19 patients who are ≥18 y of age. Nineteen trials were included in the meta-analysis. In-hospital mortality with and without vitamin C supplementation was 24.1% vs. 33.9% (OR = 0.59; 95%CI: 0.37 to 0.95; *p* = 0.03), respectively. Sub-analysis showed that, in randomized clinical trials, in-hospital mortality varied and amounted to 23.9% vs. 35.8% (OR = 0.44; 95%CI: 0.25 to 0.76; *p* = 0.003), respectively. In the non-randomized trials, in-hospital mortality was 24.2% vs. 33.5% (OR = 0.72; 95%CI: 0.38 to 1.39; *p* = 0.33), respectively. The ICU length of stay was longer in patients treated with vitamin C vs. standard therapy, 11.1 (7.3) vs. 8.3 (4.7) days (MD = 1.91; 95%CI: 0.89 to 2.93; *p* < 0.001), respectively. Acute kidney injury in patients treated with and without vitamin C varied and amounted to 27.8% vs. 45.0% (OR = 0.56; 95%CI: 0.40 to 0.78; *p* < 0.001), respectively. There were no differences in the frequency of other adverse events among patients’ treatment with and without vitamin C (all *p* > 0.05). The use of vitamin C reduces hospital mortality. The length of stay in the ICU is longer among patients treated with vitamin C. In terms of patient safety, vitamin C has an acceptable profile. Low doses of vitamin C are effective and safe. Despite some evidence of the usefulness of vitamin C in modifying the course of COVID-19, it is too early to modify guidelines and recommendations. Further studies, in particular randomized clinical trials, are necessary.

## 1. Introduction

**Figure 1:**
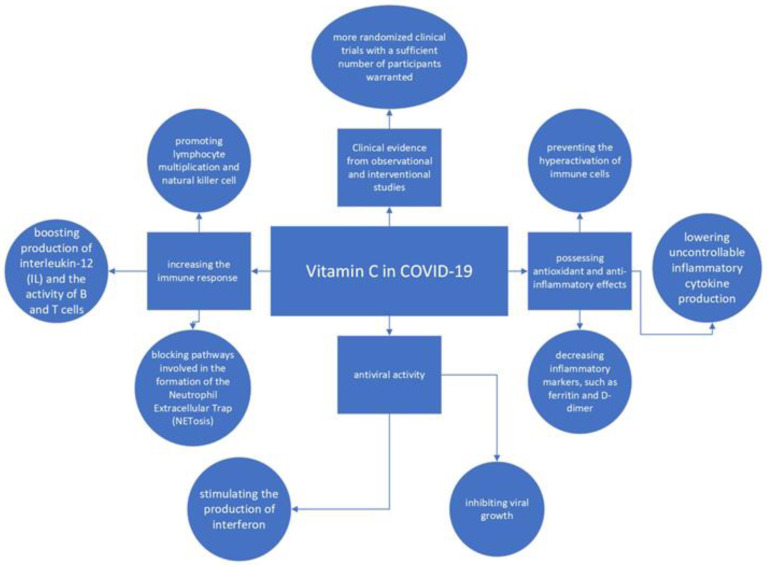
Vitamin C activities—summary.

Despite the increasingly better understanding of the pathophysiologic mechanisms of COVID-19, which translates into new therapeutic methods, the search for new, effective interventions remains a significant challenge [1,2,3]. The introduction of vaccines has not changed this need. Emerging innovative FDA and EMA-approved drugs promise to reduce patient mortality; nevertheless, many remain an expensive solution, even from the point of view of wealthy industrialized countries [4]. From the beginning of the pandemic, scientists have been inclined to study molecules with anti-inflammatory and strong antioxidant potentials. Therefore, the interest in vitamin C is not surprising [5,6], taking into consideration its characteristics. The extraordinary properties of vitamin C have been widely described in scientific literature. Above all, vitamin C is known to be a powerful antioxidant. Due to the fact that many diseases, such as cancer and coronary artery disease, can arise due to the increased activity of free radicals leading to cell and tissue damage, vitamin C as a powerful antioxidant has been studied many times in the context of its potential anti-cancer and antiatherosclerosis properties [7]. The antioxidant properties of vitamin C have led to the hypothesis of its neuroprotective properties [8]. Although some researchers also indicate the pro-oxidative effect of vitamin C, it is difficult to find unambiguous results indicating such an effect in vivo. It is even more difficult to indicate at what level of dose, administered as part of supplementation, pro-oxidative response could be manifested [9,10]. The effect of vitamin C on the immune system is complex. Stimulating the immune system with high doses of vitamin C may, paradoxically, reduce immunity. Nevertheless, in therapeutic doses, vitamin C increases immunity, e.g., due to the increased activity of neutrophils, by increasing chemotaxis and phagocytosis [11,12]. The influence on T lymphocytes and the increase in the humoral response are also important in the immunomodulatory mechanisms [13]. Vitamin C also affects the connective tissue, mainly through participation in the processes of collagen synthesis. The antiviral properties of vitamin C should be considered particularly beneficial in COVID-19 therapy. Vitamin C inhibits viral growth and stimulates the production of interferon. In addition, vitamin C enhances the antiviral activity of lung epithelial cells [14,15]. The broad activities of vitamin C that may affect its effectiveness in COVID-19 are summarized in Figure 1. From a clinical point of view, it is important to check whether there are other symptoms of vitamin C deficiency among patients with chronic wounds, especially those which are difficult to heal [16,17].

Vitamin C has been used for a long time and most often as a measure to reduce the risk of infection in the winter and autumn. However, the value of such use is highly debatable [18]. More evidence-based data are available on the use of vitamin C during the active phase of viral infection [19]. Conversely, negative health effects, visible at the population level, are observed in countries where malnutrition and inappropriate dietary habits lead to the clinical manifestation of vitamin C deficiency (Figure 1) [20]. Numerous studies show the importance of vitamin C among critically ill patients, but with an emphasis on patients with vitamin C deficiency. Vitamin C deficiency may result in an increased risk of sepsis, e.g., during pneumonia. Moreover, among patients with clinically significant vitamin C deficiency, more rapid progression of multiorgan failure in the case of sepsis diagnosis was observed [21]. The route of administration of vitamin C is also critical to its efficacy and safety. Although injections and infusions contain the highest delivered dose of vitamin C, they are also associated with the highest risk of overdose and renal side effects. Much higher doses of vitamin C administered in infusion may lead to more serious side effects resulting in severe kidney injury [22]. Determining the safety and efficacy of high-dose vitamin C has been a goal of scientific research since the beginning of the SARS-CoV-2 pandemic. Hence, the growing number of studies, including prospective evaluations, aimed at determining the usefulness of vitamin C in the primary prevention of COVID-19, preventing progression to aggressive and potentially fatal disease, as well as determining the impact of high doses of vitamin C on severely ill patients hospitalized in the ICU [23,24,25].

Thus, our purpose was to perform a meta-analysis to estimate the effect of vitamin C on in-hospital mortality and the ICU or hospital length of stay. The safety profiles between the groups of patients receiving vitamin C were also estimated.

## 2. Materials and Methods

This systematic review and meta-analysis were conducted in accordance with the Preferred Reporting Items for Systematic Reviews and Meta-analyses (PRISMA) statement [26]. The protocol of this systematic review and meta-analysis has been registered on the PROSPERO database (registration No: CRD42022349532). Ethical approval or patient consent was not suitable for this meta-analysis because all analyses were based on previously published studies.

### 2.1. Search Strategy

We conducted a literature search using PubMed, Web of Science, Scopus and Cochrane Central Register of Controlled Trials from 1 January 2020 up to 28 August 2022, to identify studies using the search terms: “Vitamin C” OR “L-Ascorbic acid” OR “Ascorbic Acid” AND

“COVID-19” OR “Corona Virus Disease 2019” OR “novel coronavirus” OR “SARS-CoV-2”. Additionally, we also manually searched the reference lists of relevant studies to identify additional relevant publications.

### 2.2. Study Selection

Two researchers (M.O.P. and M.P.) conducted data extraction independent of the included studies. Any potential discrepancies between the researchers were resolved by discussion until a consensus was reached. The final results were reviewed by the senior researcher (L.S.). The following inclusion PICOS criteria were applied for the eligible studies: (1) Patient: adult patients (aged ≥ 18 years) with COVID-19; (2) Intervention: patients treated with vitamin C supplementation; (3) Control: patients treated without vitamin C; (4) Outcomes: mortality outcomes (i.e., in-hospital mortality or 1-month mortality), hospital or ICU length of stay; (5) Study design: prospective and retrospective trials published in English. We did not define a minimum number of patients as a criterion for inclusion to be studied in our meta-analysis.

Exclusion criteria were as follows: (1) studies involving data from pediatric patients; (2) case reports, editorials, conference papers and reviews; (3) studies not in humans; (4) studies lacking research indicators required for meta-analysis; (5) studies not in English.

### 2.3. Data Extraction

Using a standard data extraction form, two researchers (M.O.P. and F.C.) performed data extraction, which was cross-verified by a third researcher (L.S.). Extracted data included the following: (1) First author’s last name, publication year and study design, route of vitamin C administration; (2) Characteristics of included studies: number of patients, age, sex (male); (3) Outcomes (mortality outcomes, ICU or hospital length of stay),: adverse events occurrence (i.e., acute kidney injury or liver injury occurrence).

### 2.4. Assessment of Risk of Bias in Included Studies

Two reviewers (M.P. and J.R.L.) independently assessed the individual studies for risk of bias using a revised tool for risk of bias in randomized trials (RoB-2 tool) [27] and a tool to determine the risk of bias in non-randomized studies of interventions (ROBINS-I tool) [28] depending on the research design. For the RoB-2 tool we assessed the following biases: (1) arising from the randomization process; (2) due to deviations from the intended intervention; (3) due to missing outcome data; (4) in the measurement of the outcome; (5) bias in the selection of the reported result. In contrast, in the case of the ROBINS-I tool, the following domains were assessed: (1) bias due to confounding; (2) bias due to the selection of participants; (3) bias in the classification of intervention; (4) bias due to deviations from the intended interventions; (5) bias due to missing data; (6) bias in the measurement of outcomes; (7) bias in the selection of the reported result. We use the RobVis application for visualizing the risk-of-bias assessments [29].

### 2.5. Statistical Analysis

Data analysis was performed using the Review Manager 5.4 software (The Nordic Cochrane Center, The Cochrane Collaboration, 2014, Copenhagen, Denmark) and the STATA 14 software (StataCorp LLC, College Station, TX, USA). Dichotomous data were presented as forest plots using odds ratios (ORs) with 95% confidence intervals (CIs). We expressed continuous variables as a mean difference (MD) with 95%CI. When data were reported as median with interquartile range, estimated means and standard deviations with the formula described by Hozo were used [30]. For quantifying statistical heterogeneity, Cochran’s Q test and I^2^ statistics were applied. An I^2^ value grouped in intervals: 0–25%, 26–50%, 51–75% and >75% represented insignificant, low, moderate and high heterogeneity, respectively [31]. The random-effects model was used for I^2^ > 50%; otherwise, the fixed-effects model was employed. Egger’s test and funnel plots were used to assess potential bias and perform funnel-plot tests for asymmetry to investigate potential publication bias if there were more than 10 trials in a single meta-analysis.

## 3. Results

### 3.1. Literature Search

**Figure 2:**
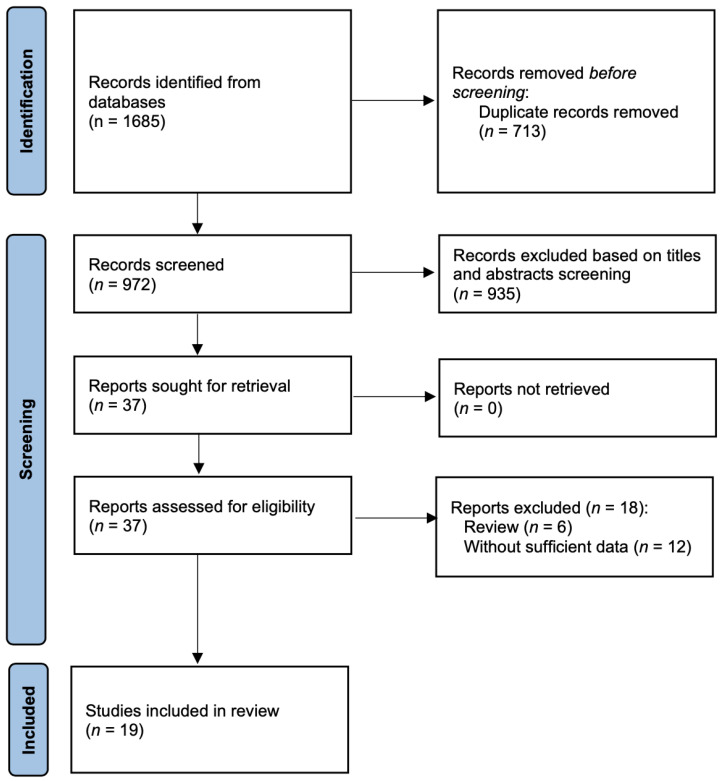
PRISMA flow diagram of the study selection process.

In summary, the search retrieved 1685 records from electronic databases. After duplicates were removed, the title and abstract of 972 records were screened by applying the inclusion/exclusion criteria previously described. The full text of thirty-seven potentially eligible articles was assessed. Nineteen studies [32,33,34,35,36,37,38,39,40,41,42,43,44,45,46,47,48,49,50] finally met relevant standards and the screening process is shown in Figure 2.

### 3.2. Trials Characteristics and Risk of Bias Assessment

The characteristics of the 19 retrieved trials were reported in Table 1. Ten studies were designed as randomized controlled trials [33,34,38,40,46,47,48]. Fourteen trials concerned intravenous vitamin C supplementation [32,34,35,36,37,39,40,41,43,44,45,46,48,49,50]. Of the 19 trials, six were performed in Iran [33,35,38,43,46], four in the USA [34,39,41,47], four in China [36,48,49,50], two in Turkey [44,45] and one in each of the following countries: Saudi Arabia [32], Pakistan [40] and Greece [37]. All included trials had a low risk of bias. The evaluation of the risk of bias in the selected studies is presented in Supplementary Figures S1–S4.

### 3.3. Data Analysis

**Figure 3:**
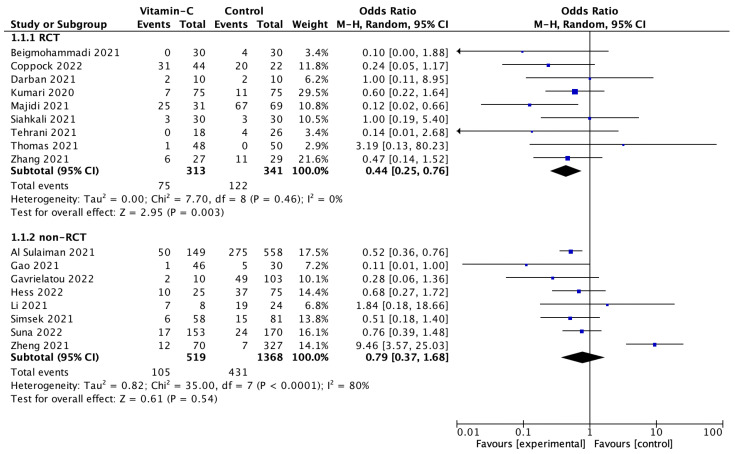
Forest plot of in-hospital mortality among COVID-19 patients with and without vitamin C supplementation. The center of each square represents the odds ratios for individual trials, and the corresponding horizontal line stands for a 95% confidence interval. The diamonds represent pooled results [32,33,34,35,36,37,39,40,41,42,43,44,45,46,47,48,50].

A total of 17 studies reported in-hospital mortality in COVID-19 patients with and without vitamin C supplementation. Pooled analysis showed that in-hospital mortality was 21.6% for patients treated with vitamin C, compared to 32.4% for a standard treatment group (OR = 0.61; 95%CI: 0.37 to 1.02; *p* = 0.06). Sub-analysis relating to the study design showed that, in randomized clinical trials, in-hospital mortality among patients treated with and without vitamin C supplementation varied and amounted to 23.9% vs. 35.8%(OR = 0.44; 95%CI: 0.25 to 0.76; *p* = 0.003; Figure 3), respectively. In the non-randomized trials, in-hospital mortality was 20.3% vs. 31.5%(OR = 0.79; 95%CI: 0.37 to 1.68; *p* = 0.54), respectively.

Additionally, a sub-analysis was performed concerning the route of vitamin C administration. In-hospital mortality in intravenous vitamin C supplementation compared to the group not treated with vitamin C was 13.0% vs. 18.9% (OR = 0.69; 95%CI: 0.36 to 1.33; *p* = 0.27), respectively. For orally administered vitamin C, the pooled analysis found a survival trend, which showed a significant reduction in in-hospital mortality of 39.3% vs. 51.8% (OR = 0.38; 95%CI: 0.17 to 0.89; *p* = 0.03), respectively.

In turn, in-hospital mortality in the group of studies in which vitamin C was used without co-supplementation with other molecules [28,29,30,31,32,34,36,37,39,40,41,42,43,44,45,46] was 20.7% and 37.4%, respectively, for patients treated without vitamin C (OR = 0.50; 95%CI: 0.39 to 0.66; *p* < 0.001). In turn, in the group of studies where vitamin C has been supplemented with other molecules [33,35,38,47], pooled analysis of in-hospital mortality was 27.4% vs. 21.2% (OR = 1.39; 95%CI: 0.25 to 7.77; *p* = 0.71), respectively.

Additionally, one-month mortality, reported in two trials, was 26.6% among patients treated with vitamin C, and 43.8% for the standard therapy group (OR = 0.48; 95%CI: 0.33 to 0.70; *p* < 0.001).

**Figure 4:**
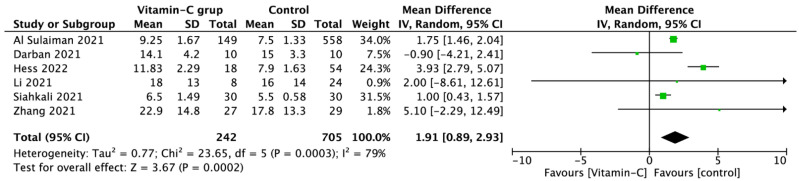
Forest plot of Intensive Care Unit length of stay among COVID-19 patients with and without vitamin C supplementation. The center of each square represents the mean differences for individual trials, and the corresponding horizontal line stands for a 95% confidence interval. The diamonds represent pooled results [32,35,39,41,43,48].

Six studies reported the ICU length of stay, which was longer in patients treated with vitamin C vs. standard therapy, 11.1 (7.3) vs. 8.3 (4.7) days (MD = 1.91; 95%CI: 0.89 to 2.93; *p* < 0.001), respectively, Figure 4.

**Figure 5:**
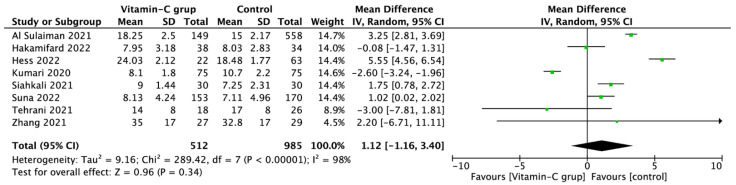
Forest plot of hospital length of stay among COVID-19 patients with and without vitamin C supplementation. The center of each square represents the mean differences for individual trials, and the corresponding horizontal line stands for a 95% confidence interval. The diamonds represent pooled results [32,38,39,40,43,45,46,48].

Pooled analysis of hospital LOS showed no differences in those treated with vitamin C vs standard therapy, 12.6 (8.7) vs 13.5 (6.5) days (MD = 1.12; 95%CI: −1.16 to 3.40; *p* = 0.34), respectively, Figure 5.

Table 2 showed adverse events among groups with and without vitamin C supplementation. Pooled analysis of three trials showed that acute kidney injury in patients treated with and without vitamin C varied and amounted to 27.8% vs. 45.0% (OR = 0.56; 95%CI: 0.40 to 0.78; *p* < 0.001), respectively. There were no differences in the frequency of other adverse events among patients’ treatment with and without vitamin C (all *p* > 0.05).

Table 3 showed that only low doses of vitamin C reduced one-month mortality (OR = 0.45; 95%CI: 0.32 to 0.64; *p* < 0.001).

## 4. Discussion

Our meta-analysis showed that the use of vitamin C in randomized trials was associated with a significant reduction in in-hospital mortality in patients with COVID-19, compared to the group of patients who did not receive vitamin C (23.9% vs. 35.8%, respectively, *p* = 0.003). However, in the case of retrospective studies, although in-hospital mortality showed a slippery trend with vitamin C supplementation, compared to the group without vitamin C supplementation (24.2% vs. 33.5%, respectively), these differences were not statistically significant. There was also no statistically significant difference in the use of vitamin C in the case of the intravenous route of administration. The same conclusions can be observed in the case of the oral formulations of vitamin C.

Only two studies estimated three-month mortality; thus, these findings are less meaningful. Conversely, vitamin C used in the ICU was associated with the opposite findings, including prolonged hospital stay. The reason behind this tendency is unknown but may be a function of the critical condition of ICU patients. Moreover, the admission criteria to the ICU may differ, sometimes significantly, between countries and sites; thus, this finding remains of undetermined importance in our analysis. In addition, it can be assumed that different research methodologies may influence such a result. A bacterial infection (secondary to the COVID-19 viral infection) can already be identified in some patients admitted to the ICU, which may additionally explain the difference in the length of stay at the ICU. It must not be forgotten that clinical practice shows that vitamin C is administered intravenously in large doses in ICU departments. This mode of administration (route of administration and dose) may be associated with an increased risk of side effects. In addition, similar observations were seen in a study that aimed to evaluate the use of intravenous vitamin C in adults with sepsis who were receiving vasopressor therapy in the intensive care unit (ICU) [51]. The increased risk of side effects may also explain why higher doses did not reduce in-hospital mortality. Moreover, if the earlier lower doses were not successful, clinicians could increase the doses of vitamin C in order to obtain the desired therapeutic effect. Thus, higher doses could be used in patients not responding sufficiently to previous treatment. The use of vitamin C with other supplementation (including zinc and vitamin D) did not reduce in-hospital mortality. This can be explained by the fact that the clinical condition of patients who received multicomponent therapy could be even worse initially than in patients who received monotherapy. It seems that the potential interaction between the components of complex therapy is not the reason for these differences, as vitamin C does not interact with zinc or vitamin D3—the most popular components of complex therapy.

Overall, the safety profile of vitamin C is highly acceptable. The frequency and severity of adverse events are in line with Reference Safety Information widely known for products containing vitamin C. Moreover, there is no statistical significance between the two groups in terms of the safety profile. As is frequently expected with severe COVID-19, respiratory failure requiring mechanical ventilation may occur. In our opinion, respiratory failure should not be reported as an adverse event associated with vitamin C treatment; nevertheless, these events were identified by some authors and, thus, were included in our study. Finally, it must be stated that the term “standard treatment” reflects heterogeneous concepts due to amendments in official guidelines within the investigated period and how that affects our results is uncertain. To sum up, a meta-analysis showed a reduction in mortality only with low doses of vitamin C. Thus, the administration of low doses of vitamin C is associated with the greatest efficacy and safety profile.

Pedrosa et al., stated that, based on pharmacological and preclinical features, vitamin C and other potent antioxidants (e.g., vitamin D) should be an effective treatment both in symptomatic COVID-19 and in primary preventive strategies [52]. Of note, vitamin C will be highly effective among patients with deficiency (hypovitaminosis C) [53]. Despite a limited number of randomized clinical trials, and conflicting results from some, Borges et al. found vitamin C as a likely beneficial adjuvant treatment for COVID-19 patients, particularly due to a reduction of inflammation and better oxygen functioning when vitamin C is provided [54]. The same conclusion is suggested by Vollbracht and Kraft, who stressed the role of vitamin C in Long-COVID. Vitamin C may greatly contribute to regulating immune response and decreasing chronic inflammation, both factors that may contribute to the development and maintenance of Long-COVID [55]. On the other hand, some authors recommended providing additional supportive nutrition only to those patients among whom hypovitaminosis was clinically demonstrated [56].

Although intravenous vitamin C may be more effective compared to oral formulations, the results of another meta-analysis suggested that a short course of intravenous vitamin C did not reduce the mortality rate or severity of COVID-19 [57]. However, only seven studies were included in the aforementioned meta-analysis. Gavrielatou et al. revealed that the use of vitamin C in the ICU has no impact on mortality rate, at least in the short course of treatment, which is in line with our findings [37]. Moreover, Migliorini et al. stated that it is too soon to make any evidence-based recommendations regarding the role of vitamin C in COVID-19 treatment [58]. A meta-analysis of randomized clinical trials showed that vitamin C did not affect mortality, the ICU length of stay, or the need for invasive mechanical ventilation. A limitation of the conclusion is the fact that our knowledge of COVID-19 treatments has increased significantly since 2021 [59]. It is worth noting that in 2021 some authors were more optimistic about the role of vitamin C in the treatment of COVID-19, especially in terms of shortening the stay in the ICU department [60]. Similarly, meta-analysis from 2022 (August) highlighted the importance of vitamin C in COVID-19, emphasizing the reduction of mortality risk among COVID-19 patients (OR = 0.54, 95%CI = 0.42–0.69, *p* < 0.00001) and indicating that patients who consumed vitamin C showed lower risk of severe COVID-19 than those who did not take vitamin C (OR = 0.63, 95%CI = 0.43–0.94, *p* = 0.02) [61]. Due to the low risk associated with an increased supply of vitamin C, from the public health point of view, its administration should at least be considered [62].

The route of administration and the concentration seem to be critical in the efficacy of vitamin C treatment. Thus, it should be of no surprise that some scientists have reported an alternative formulation. By using inhaled formulations that deliver vitamin C directly to the lungs, we may achieve an immediate local high concentration of vitamin C. On the other hand, it would be difficult, but not impossible, to achieve an acceptable size of particles, which is a critical parameter considering the design of inhalators [63].

In clinical practice, the potential of vitamin C overdosage must be considered. In the case of vitamin C, this risk is of less consequence compared to other experimental COVID-19 nutritional agents (e.g., zinc). Nevertheless, clinicians should be cautious while treating patients diagnosed with chronic renal disease because vitamin C is excreted solely by the kidneys [64].

Compared to previous meta-analyses, the present article includes more articles and more sub-analyses, e.g., the effect of vitamin C dose (low, moderate, high dose), a comparison of vitamin C used in monotherapy and combination therapies, depending on the formulation (a solid dosed form of the drug versus infusion). In addition, a meta-analysis is based on two hard endpoints, in-hospital mortality and the ICU stay, which are useful in guideline development and patient treatment planning. When pointing to the limitations of our meta-analysis, we must stress the high heterogeneity of the publications included in the analysis. Another limitation is the definition and the scope of the therapeutic standard: a so-called evolving standard of care, which has changed over the course of the development of knowledge about COVID-19 and varies depending on the location of the hospital (geographical variability). Moreover, some studies not only assessed the effectiveness of vitamin C in monotherapy, but also took into consideration combination therapies, e.g., administering vitamin C, melatonin and zinc simultaneously [35]. The development and emergence of new studies, in particular randomized clinical trials, justify further meta-analyses, including revisions of the existing ones. As for the guidelines for further studies, it seems necessary to increase the number of participants in randomized clinical trials so that the clinical trials are not pilot in nature. If it were considered ethical, the use of a placebo, even as an add-on, should be considered highly desirable.

## 5. Conclusions

The use of vitamin C reduces hospital mortality. Paradoxically, the length of stay in the ICU is longer among patients treated with vitamin C. In terms of patient safety, vitamin C has an acceptable profile. Low doses of vitamin C are effective and safe. Despite some evidence of the usefulness of vitamin C in modifying the course of COVID-19, it is too early to modify guidelines and recommendations. Further studies, in particular randomized clinical trials, are necessary.

## Figures and Tables

**Table 1:** Characteristics of included trials.

Study	Country	Study Design	Rout of Vitamin C Administration	Group with Vitamin C supplementation			Group without Vitamin C Supplementation		
				No. of Patients	Age	Sex, Male	No. of Patients	Age	Sex, Male
Al Sulaiman et al., 2021 [32]	Saudi Arabia	Retrospective study	P.O.	158	60.5 ± 15.09	124 (79.0)	581	60.7 ± 14.75	407 (70.1)
Beigmohammadi et al., 2021 [33]	Iran	RCT	P.O.	30	51.00 ± 17.25	15 (50.0%)	30	53.0 ± 7.00	16 (53.3%)
Coppock et al., 2022 [34]	USA	RCT	I.V.	44	60 ± 17	22 (50.0%)	22	61 ± 11	11 (50.0%)
Darban et al., 2021 [35]	Iran	RCT	I.V.	10	NS	NS	10	NS	NS
Gao et al., 2021 [36]	China	Retrospective study	I.V.	46	62.75 ± 4.25	21 (45.7%)	30	57.5 ± 4.5	14 (46.7%)
Gavrielatou et al., 2022 [37]	Greece	Retrospective study	I.V.	10	68.5 ± 4.9	7	103	67.5 ± 3.7	78
Hakamifard et al., 2022 [38]	Iran	RCT	P.O.	38	35.68	24 (63.2%)	34	37.41	22 (64.7%)
Hess et al., 2022 [39]	USA	Retrospective study	I.V.	25	58.3 ± 14.2	13 (52.0%)	75	71.2 ± 13.0	42 (56.0%)
Kumari et al., 2020 [40]	Pakistan	RCT	I.V.	75	52 ± 11	NS	75	53 ± 12	NS
Li et al., 2021 [41]	USA	Retrospective study	I.V.	8	64.1 + 8.3	3	24	64.9 + 11.8	9
Majidi et al., 2021 [42]	Iran	RCT	P.O.	31	59.42 ± 15.07	19	69	63.82 ± 14.58	41
Siahkali et al., 2021 [43]	Iran	RCT	I.V.	30	57.53 ± 18.27	15	30	61 ± 15.9	15
Simsek et al., 2021 [44]	Turkey	Retrospective study	I.V.	58	56.53 ± 18.77	27	81	62.20 ± 15.72	52
Suna et al., 2022 [45]	Turkey	Retrospective study	I.V.	153	60.16 ± 13.65	102	170	64.27 ± 14.49	102
Tehrani et al., 2021 [46]	Iran	RCT	I.V.	18	58 ± 19	8	26	61 ± 17	18
Thomas et al., 2021 [47]	USA	RCT	P.O.	48	45.6 ± 15.0	15	50	42.0 ± 14.6	19
Zhang et al., 2021 [48]	China	RCT	I.V.	27	66.3 ± 11.2	15 (55.6%)	29	67.0 ± 14.3	22 (75.9%)
Zhao et al., 2021 [49]	China	Retrospective study	I.V.	55	37.5 ± 4.0	33	55	37.25 ± 3.75	35
Zheng et al., 2021 [50]	China	Retrospective study	I.V.	73	67.5 ± 2.0	38 (51.1%)	323	67.5 ± 2.0	207

Legend: I.V. = intravenous; NS = not specified; P.O. = per os.

**Table 2:** Pooled analysis of adverse events.

Adverse Event Type	No of Studies	Events/Participants		Events		Heterogeneity between Trials		*p*-Value for Differences across Groups
		Vitamin C Supplementation	Standard Treatment	OR	95%CI	*p*-Value	I^2^ Statistic	
Acute kidney injury	3	67/241 (27.8%)	306/680 (45.0%)	0.56	0.40 to 0.78	0.34	7%	<0.001
Liver injury	2	26/183 (14.2%)	76/597 (12.7%)	0.83	0.49 to 1.41	0.72	0%	0.49
Respiratory failure required MV	3	50/185 (27.0%)	62/260 (23.8%)	1.22	0.78 to 1.89	0.29	18%	0.38
Coagulation disorders	2	18/181 (9.9%)	71/594 (12.0%)	0.80	0.25 to 2.49	0.09	65%	0.15
Acute cardiac injury	1	7/27 (25.9%)	13/29 (44.8%)	0.43	0.14 to 1.33	NA	NA	0.14

Legend: CI: confidence interval; NA: not applicable; OR: odds ratio.

**Table 3:** The dose of vitamin C and one-month mortality.

Vitamin C Daily Dose	No of Studies	Events/Participants		Events		Heterogeneity between Trials		*p*-Value for Differences across Groups
		Vitamin C Supplementation	Standard Treatment	OR	95%CI	*p*-Value	I^2^ Statistic	
≤1 g/day	4	108/234 (46.2%)	411/752 (54.7%)	0.45	0.32 to 0.64	0.28	22%	<0.001
>1 g/day ≤10 g/day	9	38/418 (9.1%)	72/445 (16.2%)	0.62	0.40 to 0.96	0.48	0.48	0.03
>10 g/day	4	34/180 (18.9%)	70/512 (13.7%)	1.13	0.27 to 4.73	<0.001	88%	0.87

Legend: CI: confidence interval; OR: odds ratio.

## Data Availability

The data that support the findings of this study are available on request from the corresponding author (L.S.).

## Supplementary Materials

The following supporting information can be downloaded at: https://www.mdpi.com/article/10.3390/nu14194217/s1, Figure S1: A summary table to review authors’ judgements for each risk of bias item for each randomized study. Figure S2: A plot of the distribution to review authors’ judgements across randomized studies for each risk of bias item. Figure S3: A summary table to review authors’ judgements for each risk of bias item for each non-randomized study. Figure S4: A plot of the distribution to review authors’ judgements across non-randomized studies for each risk of bias item.
