# Supplementary material for: Vitamin C Supplementation for the Treatment of COVID-19: A Systematic Review and Meta-Analysis
Source: Nutrients. 2022 Oct 10;14(19):4217. doi: 10.3390/nu14194217 (PMC9570769; doi:10.3390/nu14194217)
Supplement: Supplementary file 1 [file nutrients-14-04217-s001.zip › nutrients-1939318-supplementary.pdf]

Vitamin C Supplementation for the Treatment of COVID-19: A Systematic Review and Meta-Analysis

CONTENT

Figure S1. A summary table of review authors' judgements for each risk of bias item for each randomized study. 2

Figure S2. A plot of the distribution of review authors' judgements across randomized studies for each risk of bias item. 2

Figure S3. A summary table of review authors' judgements for each risk of bias item for each non-randomized study. 3

Figure S4. A plot of the distribution of review authors' judgements across non-randomized studies for each risk of bias item. 3

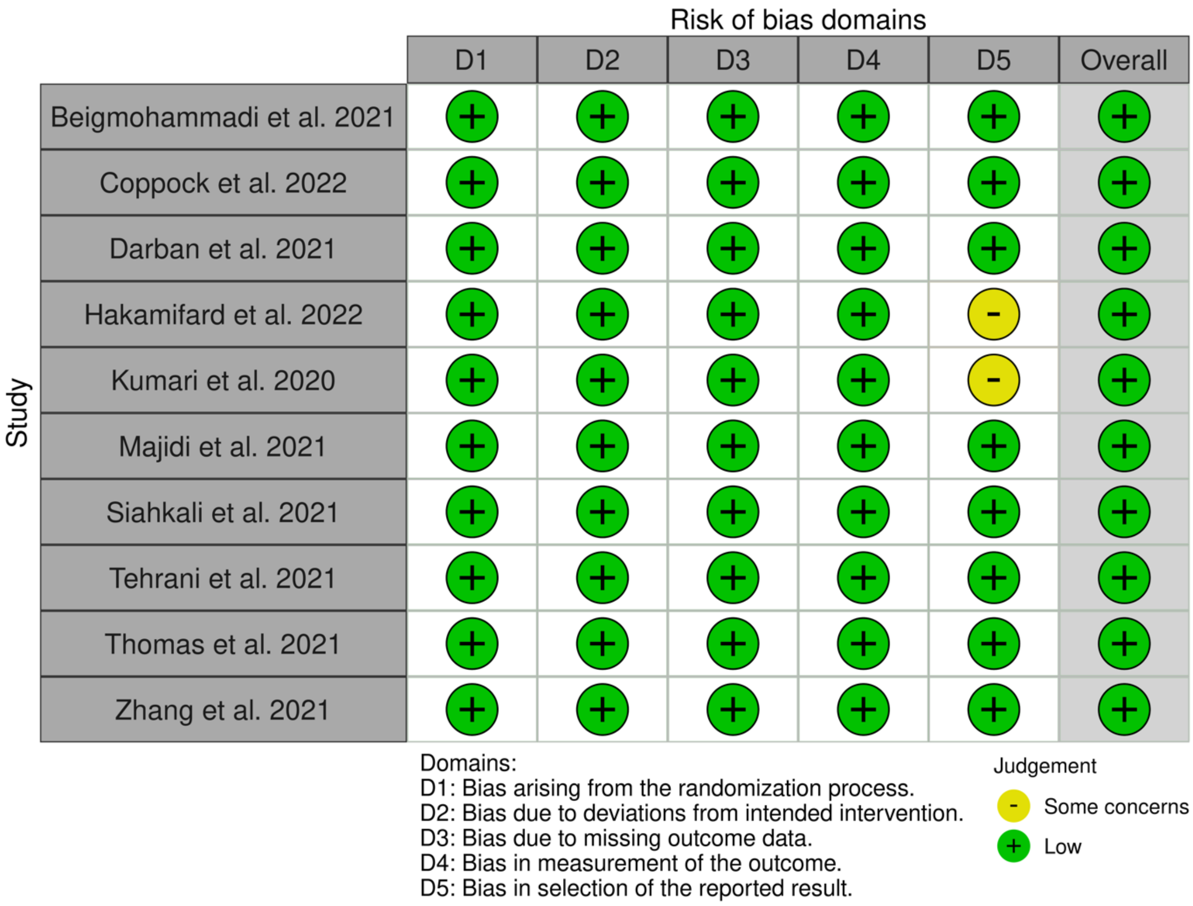

Figure S1. A summary table of review authors' judgements for each risk of bias item for each randomized study.

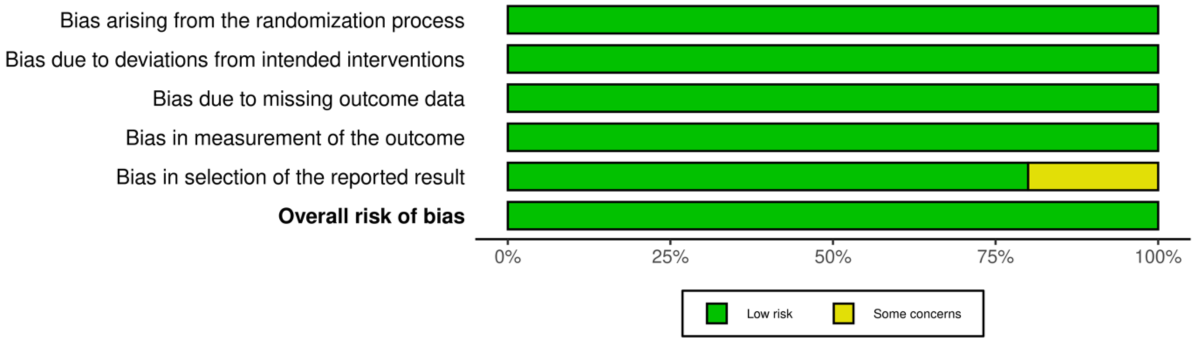

Figure S2. A plot of the distribution of review authors' judgements across randomized studies for each risk of bias item.

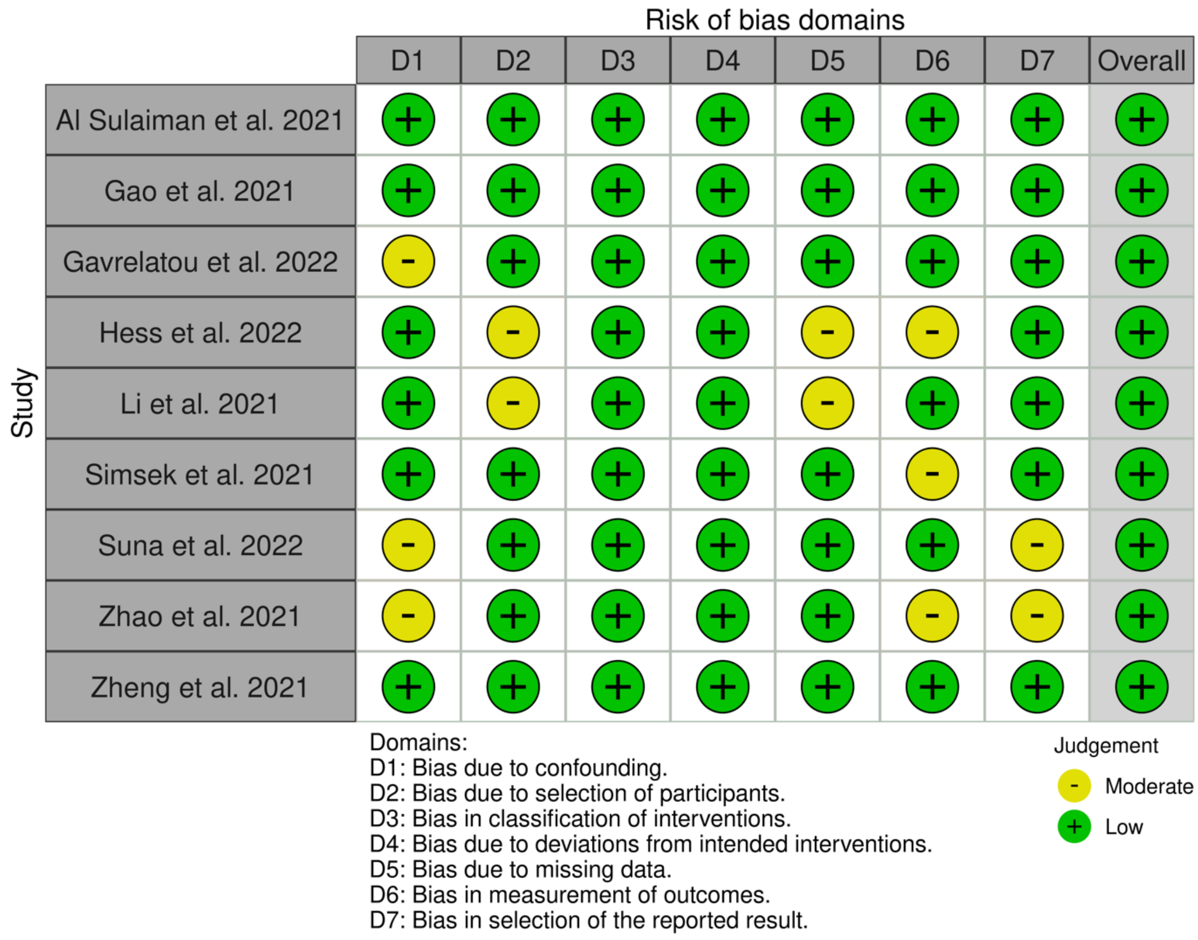

Figure S3. A summary table of review authors' judgements for each risk of bias item for each non-randomized study.

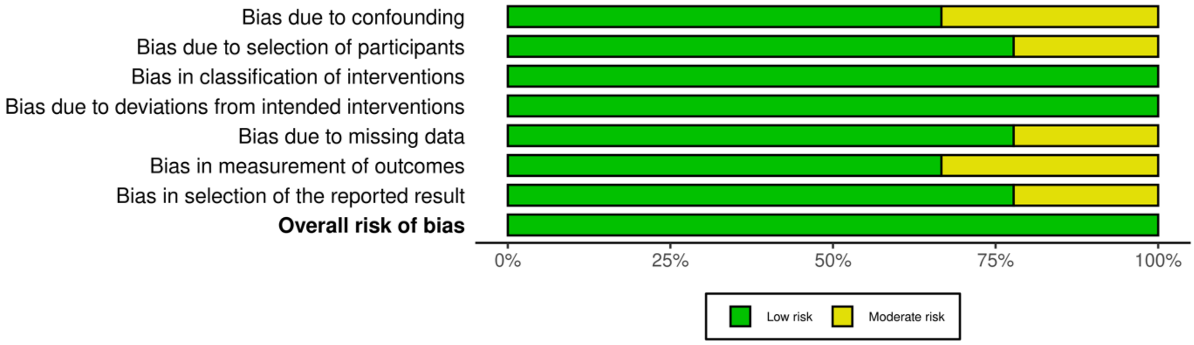

Figure S4. A plot of the distribution of review authors' judgements across non-randomized studies for each risk of bias item.
